# Manipulation of Majorana bound states in proximity to a quantum ring with Rashba coupling

**DOI:** 10.1038/s41598-022-05043-y

**Published:** 2022-01-20

**Authors:** Fabián Gonzalo Medina, Dunkan Martínez, Álvaro Díaz-Fernández, Francisco Domínguez-Adame, Luis Rosales, Pedro A. Orellana

**Affiliations:** 1 Departamento de Física, Universidad Técnica Federico Santa María, Casilla 110 V, Valparaíso, Chile; 2 GISC, Departamento de Física de Materiales, Universidad Complutense, 28040 Madrid, Spain; 3 GISC, GSC, Departamento de Estructuras y Física de Edificación, Universidad Politécnica de Madrid, 28031 Madrid, Spain

**Keywords:** Physics, Condensed-matter physics

## Abstract

The quest for Majorana zero modes in the laboratory is an active field of research in condensed matter physics. In this regard, there have been many theoretical proposals; however, their experimental detection remains elusive. In this article, we present a realistic setting by considering a quantum ring with Rashba spin-orbit coupling and threaded by a magnetic flux, in contact with a topological superconducting nanowire. We focus on spin-polarized persistent currents to assess the existence of Majorana zero modes. We find that the Rashba spin-orbit coupling allows for tuning the position of the zero energy crossings in the flux parameter space and has sizable effects on spin-polarized persistent currents. We believe that our results will contribute towards probing the existence of Majorana zero modes.

## Introduction

Majorana fermions remain a theoretical construct in the realm of particle physics. These particles, whose main feature is to be their own antiparticles, have been sought after in neutrinos, although without success thus far^1,2^. In condensed matter physics, Majorana quasiparticles emerge in *p*-wave topological superconductors^3,4^. Of particular relevance are the so-called Majorana zero modes (MZM)^2^, which are zero-energy Majorana quasiparticles protected by particle-hole symmetry. These quasiparticles, in contrast to their particle counterparts, exhibit yet another crucial feature. Such property is their being non-Abelian under braiding, which renders these quasiparticles as ideal candidates to perform topological quantum computing^5,6^. The reason for this stems in the large degeneracy found in the ground state of a system comprising a large number of Majorana quasiparticles. This degeneracy occurs due to these zero modes being pinned to zero energy^4^.

The archetypal model for studying Majorana modes in superconductors is that of Kitaev^7–9^, where a one-dimensional spinless *p*-wave superconductor is considered. Under suitable tuning of parameters, the model predicts that an ordinary superconductor undergoes a topological quantum phase transition into a topological superconductor. When considering open boundary conditions, the model predicts an odd number of MZMs at both ends of the chain with exponential decay into the bulk. Kitaev’s model, although simple, poses significant challenges for its experimental realization. However, a great deal of experimental advances have taken place during the last few years, and a convenient setting has been put forward^4,10–12^. Such a setting has three key ingredients: a semiconductor nanowire with large spin-orbit coupling, such as InAs and InSb, a uniform magnetic field parallel to the wire to induce Zeeman splitting, and an *s*-wave superconductor such as Al. When placed in contact with the semiconductor and applying the magnetic field, the superconductor becomes topological when a given magnetic field is reached.

In this paper, we consider a superconducting nanowire in the topological regime placed in contact to a quantum ring. We assume the ring to be a continuation of the nanowire, as was done with a quantum dot in Ref.^13^, and therefore it displays strong spin-orbit coupling. The ring is in fact a collection of quantum dots assembled in a ring-like fashion, which can be achieved by means of gate voltages. The main results of this paper relate to the impact of the nanowire on the persistent spin currents of the ring, which are sharply different from those of the ring. As we shall show, when the MZMs interact via the quantum ring, they split from zero energy and oscillate, forming zero energy crossings. These crossings reshape the spin currents that would be present in the absence of Majoranas. Therefore, we believe our proposal could potentially be used as an additional tool to uncover the nature of MZMs.

## System and model Hamiltonian

**Figure 1:**
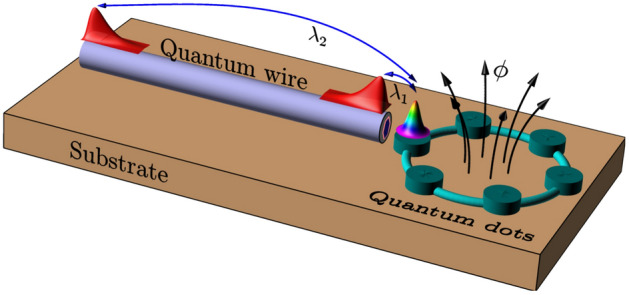
(**a**) Schematic representation of the system under study. A nanowire driven into a superconducting regime supports a MZM at each edge and influences the persistent currents of a quantum ring, threaded by a magnetic flux. (**b**) The quantum ring consists of an array of quantum dots uniformly distributed on a circumference. The MZMs are assumed to be coupled to the same quantum dot of the ring, with coupling constants $\lambda _1$ and $\lambda _2$.

The system under consideration consists of a topological superconducting nanowire hosting MZMs at its edges, as depicted in Fig. 1a. The nanowire is placed in close proximity to a quantum ring, which is threaded by a magnetic flux. We introduce a minimal model that captures the influence of the MZMs on the persistent currents of the ring. The quantum ring consists of an array of quantum dots uniformly distributed on a circumference, as shown in Fig. 1b. Due to the similarities of our system with the one considered in Refs.^12,13^, we believe that our model might be amenable to be performed experimentally.

The model Hamiltonian is then given by

1 $$\begin{aligned} H = H_r + H_M + H_c \ , \end{aligned}$$

where $H_r$ is the Hamiltonian of the ring, $H_M$ describes the interaction between the two MZMs at the edges of a nanowire in the topological superconducting regime, and $H_c$ contains the coupling between the MZMs and the quantum ring. Considering spin-orbit coupling and the fact that the quantum ring is threaded by a flux $\Phi$, $H_r$ can be written as^14–16^

2 $$\begin{aligned} H_{r} = - \sum _{n}\left[ {\varvec{c}}_n^{\dagger }\big ({\varvec{t}}+{\varvec{t}}_\mathrm {so}(n)\big )e^{\mathrm {i}\,\theta }{\varvec{c}}_{n+1}^{}+\mathrm {h.c.}\right] \ , \end{aligned}$$

where ${\varvec{c}}_n^{\dagger }=\left( c_{n,\uparrow }^{\dagger },c_{n,\downarrow }^{\dagger }\right)$ are the creation operators of the quantum ring and *n* runs from 1 to *N*, with *N* the number of sites of the ring, that is, the number of quantum dots that compose it. We shall measure energies in units of 2*t* with *t* the hopping energy, which implies that ${\varvec{t}}=\sigma _0/2$, with $\sigma _0$ the $2\times 2$ identity matrix. The flux is accounted for in the phase factor $\theta =2\pi \Phi /N$, where $\Phi =\phi /\phi _0$ is the flux $\phi$ measured in units of the flux quantum $\phi _0=h/e$. The spin-orbit coupling is given by

3 $$\begin{aligned} {\varvec{t}}_\mathrm {so}(n) = \mathrm {i}\,\tilde{\alpha }\left( \sigma _x\cos \varphi _{n}+\sigma _y\sin \varphi _{n}\right) \ , \end{aligned}$$

where we only consider Rashba spin-orbit interaction, disregarding a possible Dresselhaus interaction^15^. Here, $\sigma _x$ and $\sigma _y$ are the Pauli matrices, $\tilde{\alpha } = \alpha N/2 \pi R$ being *R* the radius of the ring, $\alpha$ the strength of the Rashba interaction and

4 $$\begin{aligned} \varphi _{n}=\frac{2\pi }{N}\left( n-\frac{1}{2}\right) \ . \end{aligned}$$

Regarding the mutual coupling between MZMs and the coupling between MZMs and the ring, we will consider a low energy theory as in Ref.^17^. Thus, the interaction term between the two MZMs is given by the minimal Kitaev Hamiltonian for the topological phase^7^

5 $$\begin{aligned} H_M = \mathrm {i}\,\xi _M\gamma _1\gamma _2 \ , \end{aligned}$$

where $\gamma _1$ and $\gamma _2$ are Majorana operators which satisfy $\gamma _i=\gamma_{i}^{\dagger }$ and $\left\{ \gamma _i,\gamma _j\right\} =\delta _{ij}$. Here $\xi _M\propto e^{-L/\ell _0}$ where *L* is the length of the nanowire and $\ell _0$ is the superconducting coherence length^7^. The Majorana operators can also be written in terms of ordinary fermion operators, *f* and $f^{\dagger }$, as

6 $$\begin{aligned} \gamma _1 = \frac{1}{\sqrt{2}}(f+f^{\dagger }) \ , \quad \gamma _2 = \frac{\mathrm {i}\,}{\sqrt{2}}(f-f^{\dagger }) \ . \end{aligned}$$

In terms of these operators, Eq. (5) takes the form

7 $$\begin{aligned} H_M = \xi _M\left( f^{\dagger }f-\frac{1}{2}\right) \ . \end{aligned}$$

Finally, the coupling between the MZMs and the quantum ring is given by^17–19^

8 $$\begin{aligned} H_c = \lambda _1\left( c_{1,\uparrow }^{\dagger }-c_{1,\uparrow }^{}\right) \gamma _1+\mathrm {i}\,\lambda _2\left( c_{1,\uparrow }^{\dagger }+c_{1,\uparrow }^{}\right) \gamma _2 \ , \end{aligned}$$

where $\lambda _1$ and $\lambda _2$ are real parameters [see also Fig. 1]. We have chosen to couple the MZMs to a single spin. This can be achieved by fixing the spin canting angles of the MZMs accordingly, which can be done by means of sufficiently large magnetic fields^17^. Finally, we will not consider electron-electron interactions in the ring, which could be included within a mean field approximation^16,17^.

It is now convenient to turn to a representation where $H_r$ is diagonal. For that matter, we can use the following unitary transformation^15^

9 $$\begin{aligned} \mathscr {U}_n = \frac{1}{\sqrt{2}} \begin{pmatrix} 1 &{} -1 \\ e^{\mathrm {i}\,\varphi _{n}} &{} e^{\mathrm {i}\,\varphi _{n}} \end{pmatrix} \ , \end{aligned}$$

so that ${\varvec{c}}_n = \mathscr {U}_n\tilde{{\varvec{c}}}_n$. This transformation turns $H_r$ into

10 $$\begin{aligned} H_r = - \sum _{n}\left( \tilde{{\varvec{c}}}_n^{\dagger }\mathscr {M}e^{\mathrm {i}\,\theta }\tilde{{\varvec{c}}}_{n+1}^{} + \mathrm {h.c.}\right) \ . \end{aligned}$$

where

11 $$\begin{aligned} \mathscr {M} = e^{\mathrm {i}\,\pi /N} \begin{pmatrix} \left( \dfrac{1}{2}+\mathrm {i}\,\tilde{\alpha }\right) \cos \left( \dfrac{\pi }{N}\right) &{} \left( \dfrac{\mathrm {i}\,}{2}-\tilde{\alpha }\right) \sin \left( \dfrac{\pi }{N}\right) \\ \left( \dfrac{\mathrm {i}\,}{2}+\tilde{\alpha }\right) \sin \left( \dfrac{\pi }{N}\right) &{} \left( \dfrac{1}{2}-\mathrm {i}\,\tilde{\alpha }\right) \cos \left( \dfrac{\pi }{N}\right) \end{pmatrix} \ . \end{aligned}$$

Notice that the unitary transformation has eliminated the site dependence in the hopping matrix in Eq. (10). Thus, by Fourier transforming $H_r$ we can block-diagonalize it

12 $$\begin{aligned} H_r = \sum _{k}\tilde{{\varvec{c}}}_k^{\dagger }\tilde{{\varvec{h}}}_k^{}\tilde{{\varvec{c}}}_k^{} \ , \end{aligned}$$

with

13 $$\begin{aligned} \tilde{{\varvec{h}}}_k = -\cos \left( \frac{\pi }{N}\right) \cos \left( k+\theta +\frac{\pi }{N}\right) \sigma _0+\sin \left( k+\theta +\frac{\pi }{N}\right) \left[ \sin \left( \frac{\pi }{N}\right) \sigma _x-2\tilde{\alpha }\sin \left( \frac{\pi }{N}\right) \sigma _y+2\tilde{\alpha }\cos \left( \frac{\pi }{N}\right) \sigma _z\right] \ . \end{aligned}$$

In the basis that diagonalizes $\tilde{{\varvec{h}}}_k$ we can write $H_r$ as

14 $$\begin{aligned} H_r = \sum _{n,\mu =\pm }\varepsilon _{n\mu }d_{n\mu }^{\dagger }d_{n\mu }^{} \ , \end{aligned}$$

where the eigenvalues are

15 $$\begin{aligned} \varepsilon _{n,\pm } = -\cos \left( \frac{\pi }{N}\right) \cos \left[ \frac{2\pi }{N}\left( n+\frac{1}{2}+\Phi \right) \right] \pm \sin \left[ \frac{2\pi }{N}\left( n+\frac{1}{2}+\Phi \right) \right] \sqrt{\sin ^2\left( \frac{\pi }{N}\right) +4\tilde{\alpha }^2} \ . \end{aligned}$$

In this basis, $H_c$ is written as follows

16 $$\begin{aligned} H_c = \sum _{n,\mu =\pm }\Big [ \beta _{n,\mu }^{}d_{n,\mu }^{\dagger }\left( \lambda _{-}f^{\dagger }+\lambda _{+}f\right) -\beta _{n,\mu }^{*}d_{n,\mu }^{}\left( \lambda _{-}f+\lambda _{+}f^{\dagger }\right) \Big ]\ , \end{aligned}$$

where $$\lambda _{\pm } =(\lambda _1\pm \lambda _2)/2$$, $$\beta _{n,-} = e^{-\mathrm {i}\,2\pi n/N}(\psi _{-}^{u}-\psi _{+}^{u})$$, $$\beta _{n,+} = e^{-\mathrm {i}\,2\pi n/N}(\psi _{-}^{d}-\psi _{+}^{d})$$ with

17 $$\begin{aligned} \psi _{\pm }^u&= u_{\pm }/\sqrt{1+|u_{\pm }|^2} \ , \nonumber \\ \psi _{\pm }^d&= 1/\sqrt{1+|u_{\pm }|^2}\ , \nonumber \\ u_{\pm }&= \dfrac{2\tilde{\alpha }\cos \left( \frac{\pi }{N}\right) \pm \sqrt{\sin ^2\left( \frac{\pi }{N}\right) +4\tilde{\alpha }^2}}{(1-2\mathrm {i}\,\tilde{\alpha })\sin \left( \frac{\pi }{N}\right) } \ . \end{aligned}$$

We can now find the spectrum of *H* by using the Bogoliubov-de Gennes (BdG) Hamiltonian. Indeed, if we introduce

18 $$\begin{aligned} {{\varvec{\Psi }}}=\left( \left\{ d_{n,\mu }^{}\right\} ,f,\left\{ d_{n,\mu }^{\dagger }\right\} ,f^{\dagger }\right) ^{T} \ , \end{aligned}$$

with $\left\{ d_{n,\mu }^{}\right\} =d_{1,-},d_{1,+},\dots ,d_{N,-},d_{N,+}$ and likewise for $\left\{ d_{n,\mu }^{\dagger }\right\}$, we can write *H* as

19 $$\begin{aligned} H = \frac{1}{2}{{\varvec{\Psi }}}^{\dagger }\mathscr {H}_{\mathrm {BdG}}{{\varvec{\Psi }}} \ , \end{aligned}$$

where

20 $$\begin{aligned} \mathscr {H}_{\mathrm {BdG}} = \begin{pmatrix} \mathscr {H} &{} \Lambda \\ -\Lambda ^{*} &{} -\mathscr {H}^{*} \end{pmatrix} \ , \end{aligned}$$

is the BdG Hamiltonian. Here, $\mathscr {H}=\mathscr {H}_0+\mathscr {V}$ and $\Lambda$ are the following $(2N+1)\times (2N+1)$ matrices

21 $$\begin{aligned} \mathscr {H}_0= & {} \mathrm {diag}\left[ \varepsilon _{1,-},\varepsilon _{1,+},\dots ,\varepsilon _{N,-}, \varepsilon _{N,+},\xi _M\right] \end{aligned}$$

2223where $\mathbf{0}_{2N}$ is the $2N\times 2N$ zero matrix. The BdG Hamiltonian leads to a particle-hole redundancy and, as a result, the spectrum will be symmetrical around $E=0$. Diagonalization of $\mathscr {H}_{\mathrm {BdG}}$ provides us with the quasiparticle spectrum, $E(\Phi )$.

## Quasiparticle energy spectra

**Figure 2:**
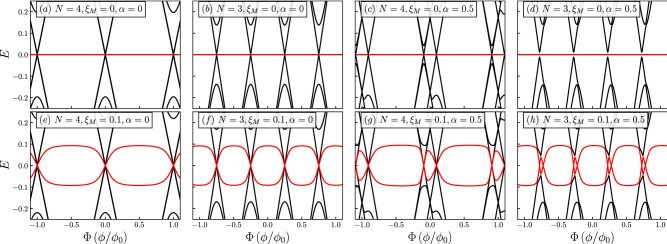
Quasiparticle spectra for $\lambda _1=0.1$ and $\lambda _2=0$. The number of sites, *N*, the coupling between MZMs, $\xi _M$, and the Rashba spin-orbit coupling, $\alpha$, are indicated in the figure. Black curves represent the states in the quantum ring and the red curves correspond to the Majorana modes.

**Figure 3:**
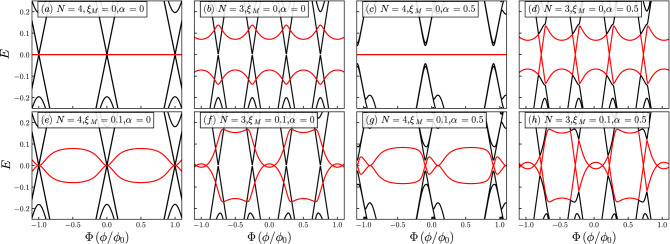
Quasiparticle spectra for $\lambda _1=0.1$ and $\lambda _2=0.15$. The number of sites, *N*, the coupling between MZMs, $\xi _M$, and the Rashba spin-orbit coupling, $\alpha$, are indicated in the figure. Black curves represent the states in the quantum ring and the red curves correspond to the Majorana modes.

In Figs. 2 and 3, we show the quasiparticle spectrum for rings with $N=3$ and $N=4$. The spectra for larger values of *N* is provided in the Supplemental Material S1, and restrict to $N=3$ and 4 in the remaining of the text. This is mainly for practical reasons, since the larger the number of dots, the harder an experimental implementation would prove to be.. In the following, we consider two scenarios, namely $\lambda _1=0.1,\lambda _2=0$ and $\lambda _1=0.1,\lambda _2=0.15$, respectively. As explained in Ref.^13^, the quantity $\eta =\sqrt{\lambda _2/\lambda _1}$ provides a measure of locality and, as reported in that reference, a value of $\eta \ll 1$ corresponds to highly nonlocal Majoranas, whereas the Majorana components of an Andreev bound state have $\eta \simeq 1$. Since in our case $\lambda _1$ and $\lambda _2$ are interchangeable, we can see that the values chosen are in the nonlocal regime, that is, $\eta <1$. In Figs. 2a,b,e,f, we consider the case of $\alpha =0$ and coupling to a single Majorana mode, namely to $\gamma _1$. In this case, we can observe that the spectrum is symmetric as $E(\Phi )=E(-\Phi )$ and the Majorana modes remain at zero energy [see panels (a) and (b)] as long as there is no coupling between them, that is, as long as $\xi _M=0$. However, the coupling to $\gamma _1$ breaks the double degeneracy that would otherwise be present in the Dirac cone-like features of the spectra, as can be observed at zero flux. Notice that the degeneracy is lifted only for the spin-up bands, as can be understood from the coupling of the MZMs to the quantum ring in Eq. (8). Nevertheless, this coupling does not lift extra degeneracies, as we shall show when adding Rashba spin-orbit coupling. Once the coupling between MZMs is introduced [see panels (e) and (f)], we see the Majorana oscillations that naturally arise in finite-sized nanowires ^2^. There are high symmetry points of the spectrum, namely at the Dirac points, where the hybridization between MZMs effectively vanishes. In panels (c), (d), (g) and (h), while the ring is still coupled to a single MZM, we introduce Rashba spin-orbit coupling. It can be observed that the effect corresponds to a lateral shift of the bands, as expected when Rashba spin-orbit interaction is present. It can be noticed, as we anticipated above, that only certain degeneracies are lifted by the coupling to the Majorana mode. Indeed, for $N=4$ we can see that the band that shifts to the left-hand side displays both gapless and gaped Dirac-like spectra close to zero energy, while the band that moves rightwards only shows a Dirac cone-like feature. However, for $N=3$ we can see that the two shifted sides present a gaped Dirac-like spectra; this is because the Rashba spin-orbit coupling makes possible the interaction between MZMs and spin down. This shift is also observed in the oscillatory behavior of the MZMs as they are coupled together. However, these shifts follow the Dirac points corresponding to the spin-up states to which $\gamma _1$ is coupled, as we can observe in panels (g) and (h).

In Fig. 3a,b,e,f, we consider the effect of coupling between the two MZMs while keeping the Rashba spin-orbit coupling turned off, $\alpha =0$. As we can observe, the degeneracy is further lifted by coupling the ring to the two MZMs, as can be observed at zero flux. However, as we expressed above, it is not entirely lifted since there are still gapless Dirac-like features at low energy. It is interesting to notice that there is a clear distinction between even- and odd-numbered rings. Indeed, even when the MZMs are explicitly uncoupled in the Hamiltonian, when $\xi _M=0$, there is hybridization due to their coupling through the ring. Although we do not show it here, this behavior can be seen to occur in rings with a larger number of sites. This effect may be due to the fact that even-numbered rings with $\alpha =0$ and at zero flux present particle-hole symmetry $c_{n,\sigma }\rightarrow (-1)^{n}c_{n,-\sigma }^{\dagger }$ and $c_{n,\sigma }^{\dagger }\rightarrow (-1)^{n}c_{n,-\sigma }$, whereas odd-numbered rings do not, making these two systems inherently different. Notice that this particle-hole symmetry is a true symmetry, whereas the particle-hole symmetry of the BdG Hamiltonian is a redundancy. Finally, in panels (c), (d), (g) and (h), we show the effect of including Rashba spin-orbit coupling, which breaks the symmetry $E(\Phi )=E(-\Phi )$ by shifting sideways the bands, as occurred in panels (c), (d), (g) and (h) of Fig. 2. However, in contrast to the latter, the coupling to $\gamma _2$ leads to a further reduction of the degeneracies in the problem. In fact, for $N=4$, the spectrum is wholly gaped out, even for $\xi _M=0$. There is then a clear distinction with the corresponding spectrum without Rashba spin-orbit coupling, which will affect the persistent currents, as we shall show below.

## Charge and spin persistent currents

Persistent currents have been studied extensively, both theoretically^16,20–25^ and experimentally^26–29^. They constitute a peculiar quantum phenomenon resulting from the time-reversal symmetry breaking that occurs from coupling electrons to a gauge field, as in the Aharonov-Bohm effect.

In this section, we will study the appearance of persistent charge, $I_q$, and spin currents, $I_s$, in all the scenarios discussed above. We will calculate $I_q$ as

24 $$\begin{aligned} I_q = -\mathrm {i}\,\left\langle \left[ N_\uparrow +N_\downarrow ,H_r\right] \right\rangle \ , \end{aligned}$$

and $$I_s$$ as

25 $$\begin{aligned} I_s = -\mathrm {i}\,\left\langle \left[ N_\uparrow -N_\downarrow ,H_r\right] \right\rangle \ , \end{aligned}$$

where $N_\sigma =\sum _{n}c_{n,\sigma }^{\dagger }c_{n,\sigma }$. Notice that the charge persistent current can also be obtained from the eigenenergies $E_{\nu }$ by summing over all states $I_{q}=\sum _{\nu }I_{q,\nu }$ where^16^

26 $$\begin{aligned} I_{q,\nu } = -\frac{\partial }{\partial \Phi }\left[ f(E_\nu )E_\nu \right] \ , \end{aligned}$$

and $f(E_\nu )$ the Fermi distribution function. The total persistent current is obtained by summing over all states ^16^. After lengthy but straightforward calculations, we find that

27 $$\begin{aligned} I_{q(s)} = -\sum _{n}\Big [\chi _n^{q(s)}+\gamma _n^{q(s)}-\chi _{n}^{q(s)}\langle d_{n,-}^{}d^{\dagger }_{n,-}\rangle - \gamma _{n}^{q(s)}\langle d_{n,+}^{}d^{\dagger }_{n,+}\rangle - 2\text {Re}\left\{ \beta ^{q(s)}_n\langle d_{n,+}^{}d^{\dagger }_{n,-}\rangle \right\} \Big ] \ . \end{aligned}$$

As we can see, the expressions for the persistent currents are identical, except for the coefficients. For the charge persistent current, we find

28 $$\begin{aligned} \chi _{n}^q&= A_{n}^{+}\left| \psi ^{u}_{-}\right| ^{2} + A_{n}^{-}\left| \psi ^{u}_{+}\right| ^{2} + 2\text {Re}\left\{ B_{n}\psi ^{u}_{-}\left( \psi ^{u}_{+}\right) ^{*}\right\} \ ,\nonumber \\ \gamma _{n}^q&= A_{n}^{+}\left| \psi ^{d}_{-}\right| ^{2} + A_{n}^{-}\left| \psi ^{d}_{+}\right| ^{2} + 2\text {Re}\left\{ B_n \psi ^{d}_{-} \left( \psi ^{d}_{+}\right) ^{*}\right\} \ , \nonumber \\ \beta ^{q}_{n}&= A_{n}^{+}\psi ^{u}_{-}\left( \psi ^{d}_{-}\right) ^{*} + A_{n}^{-}\psi ^{u}_{+}\left( \psi ^{d}_{+}\right) ^{*} + B_{n}\psi ^{u}_{-}\left( \psi ^{d}_{+}\right) ^{*} + B_{n}^{*}\psi ^{u}_{+}\left( \psi ^{d}_{-}\right) ^{*} \ , \end{aligned}$$

where

29 $$\begin{aligned} \begin{aligned} A_{n}^{\pm } =&\Bigg [\sin \left( \frac{\pi }{N} + \frac{2\pi }{N}\Phi + \frac{2\pi n}{N}\right) \pm 2\tilde{\alpha }\cos \left( \frac{\pi }{N} + \frac{2\pi }{N}\Phi + \frac{2\pi n}{N}\right) \Bigg ]\cos \left( \frac{\pi }{N}\right) \ ,\\ B_{n} =&\left( 1 +i 2\tilde{\alpha }\right) \cos \left( \frac{\pi }{N} + \frac{2\pi }{N}\Phi + \frac{2\pi n}{N}\right) \sin \left( \frac{\pi }{N}\right) \ . \end{aligned} \end{aligned}$$

The expressions for the spin persistent current coefficients are identical by replacing $\sin \leftrightarrow \cos$ in $A_{n}^{\pm }$ and $B_{n}$ and by taking $\tilde{\alpha } \rightarrow -\tilde{\alpha }$ in $B_{n}$. Considering the fluctuation-dissipation theorem, we can write

30 $$\begin{aligned} \begin{aligned} I_{q(s)} = -\sum _n \frac{1}{\pi }\intop _{-\infty }^{\infty } d\omega f\left( \omega \right) \left[ \chi _{n}^{q(s)}\text {Im}\langle \langle d_{n,-};d^{\dagger }_{n,-}\rangle \rangle _{\omega }\right. + 2\text {Re}\left\{ \beta ^{q(s)}_{n}\text {Im}\langle \langle d_{n,-};d^{\dagger }_{n,+} \rangle \rangle _{\omega }\right\} \left. + \gamma _{n}^{q(s)}\text {Im}\langle \langle d_{n,+};d^{\dagger }_{n,+}\rangle \rangle _{\omega }\right] \ . \end{aligned} \end{aligned}$$

Here $\langle \langle \dots \rangle \rangle$ stands for the Green’s functions of the ring. Notice that we have dropped the first two terms in Eq. (27), $\chi _n^{q(s)}$ and $\gamma _{n}^{q(s)}$, since they end up cancelling out. The Green’s functions can be obtained by applying the equation of motion technique^30^, which are found to be

31 $$\begin{aligned} \begin{aligned} \langle \langle d_{n,-};d^\dagger _{n,-} \rangle \rangle&= g_{p,-} + \frac{g_{p,-}^2 |\beta _{n,-}|^2}{\tilde{M}_p\tilde{M}_h - \tilde{S}^2} \left[ \lambda _-^2 \tilde{M}_p + \lambda _+^2 \tilde{M}_h + 2\lambda _+\lambda _-\tilde{S} \right] \\ \langle \langle d_{n,+};d^\dagger _{n,+} \rangle \rangle&= g_{p,+} + \frac{g_{p,+}^2 |\beta _{n,+}|^2}{\tilde{M}_p\tilde{M}_h - \tilde{S}^2}\left[ \lambda _-^2 \tilde{M}_p + \lambda _+^2 \tilde{M}_h + 2\lambda _+\lambda _-\tilde{S} \right] \ ,\\ \langle \langle d_{n,-};d^{\dagger }_{n,+}\rangle \rangle&= \frac{g_{p,-}\beta _{n-}g_{p,+}\beta ^*_{n+}}{\tilde{M}_p\tilde{M}_h - \tilde{S}^2}\left[ \lambda _-^2 \tilde{M}_p + \lambda _+^2 \tilde{M}_h + 2\lambda _+\lambda _-\tilde{S} \right] \ , \end{aligned} \end{aligned}$$

where $g_{p,\pm }=(z-\varepsilon _{n,\pm })^{-1}$ are the Green’s functions of the quantum ring in the absence of interaction with the MZMz and

32 $$\begin{aligned} \begin{aligned} \tilde{M}_{p} =&z-\xi _M - \sum _{n}\left( \lambda ^{2}_{-}S_{h} + \lambda ^{2}_{+}S_{p}\right) \ ,\\ \tilde{M}_{h} =&z+\xi _M - \sum _{n}\left( \lambda ^{2}_{+}S_{h} + \lambda ^{2}_{-}S_{p}\right) \ ,\\ \tilde{S} =&\lambda _{+}\lambda _{-}\sum _{n}\Big (\left| \beta _{n,-}\right| ^{2}g_{h,-} + \left| \beta _{n,+}\right| ^{2}g_{h,+} +\left| \beta _{n,-}\right| ^{2}g_{p,-} + \left| \beta _{n,+}\right| ^{2}g_{p,+}\Big )\ . \end{aligned} \end{aligned}$$

**Figure 4:**
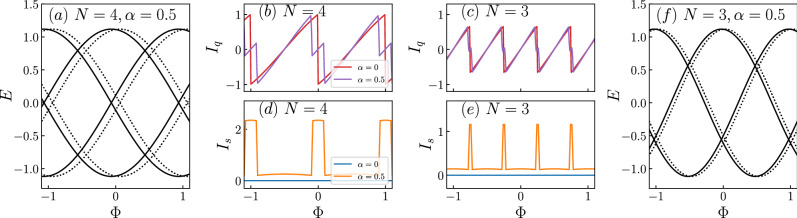
Quasiparticle spectra and corresponding charge and spin persistent currents, $I_q$ and $I_s$ respectively, for $\lambda _1=\lambda _2=\xi _M=0$. The number of sites, *N*, and the Rashba spin-orbit coupling, $\alpha$, are indicated in the figures.

Here $g_{h,\pm }=(z+\varepsilon _{n,\pm })^{-1}$ are the Green’s functions for holes of the ring uncoupled to the MZMs. In order to assess the effect of having MZMs, we first show in Fig. 4 the charge and spin persistent currents for the system without MZMs. Additionally, we include the quasiparticle spectra where it can be seen that, as expected, the only effect is a lateral shift, as indicated by solid and dashed lines. Here, panels (b) and (c) correspond to the charge persistent currents for the $N=3$ and $N=4$ cases respectively and panels (d) and (e) correspond to the spin persistent currents for the $N=3$ and $N=4$ cases respectively. The different colours indicate the value of the Rashba spin-orbit interaction. As it can be noticed, the Rashba interaction leads to a secondary peak in the charge persistent current and to nonzero spin persistent currents.

**Figure 5:**
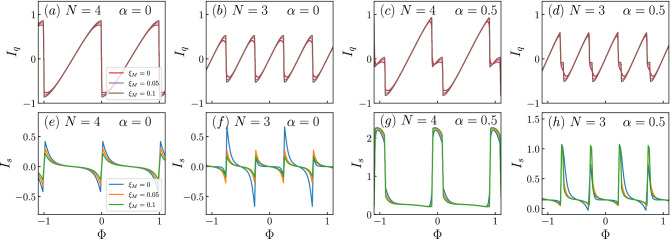
Charge and spin persistent currents, $I_q$ and $I_s$ respectively, for $\lambda _1=0.1,\lambda _2=0$. The number of sites, *N*, and the Rashba spin-orbit coupling, $\alpha$, are indicated in the figures. The coupling between MZMs, $\xi _M$, is indicated by coloured lines, shown in the leftmost panels.

**Figure 6:**
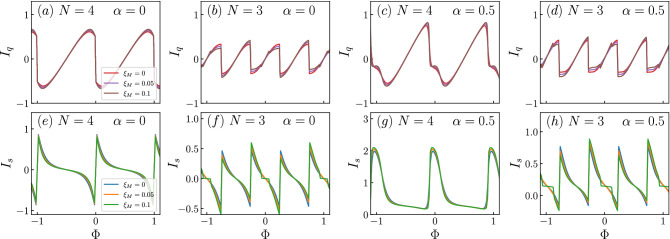
Charge and spin persistent currents, $I_q$ and $I_s$ respectively, for $\lambda _1=0.1,\lambda _2=0.15$. The number of sites, *N*, and the Rashba spin-orbit coupling, $\alpha$, are indicated in the figures. The coupling between MZMs, $\xi _M$, is indicated by coloured lines, shown in the leftmost panels.

We now study the persistent currents for the cases discussed in Figs. 2, 3 and obtain Figs. 5, 6. Here we use the same labeling as in the last figure, but the colours indicate three different values of $\xi _M$, one being $\xi _M=0$ [panels (a)–(d) in the BdG spectra], another being $\xi _M=0.1$ [panels (e)–(h) in the BdG spectra] and another one in between, $\xi _M=0.05$, whose spectrum is not shown.

Figure 5a,b,e,f corresponds to the settings of Fig. 2a,b,e,f. The first thing we observe is that the persistent currents display the periodicity observed in the spectra, as it should. In the case of the charge currents, we can see that upon decreasing the interaction between the two MZMs, the peaks in the current become smoother. On the other hand, the spin currents are nonzero due to the coupling of one of the two MZMs to the up-spin electrons in the ring. However, as the coupling between MZMs increases, reducing their topological robustness, the spin current tends towards zero. This is consistent with the results shown in the Fig. 4 as a greater interaction hybridizes the Majoranas.

Figure 5c,d,g,h refers to the scenario considered in Fig. 2c,d,g,h, which is identical to the previous one except for a nonzero Rashba spin-orbit coupling. As we can see, in this case, the charge current is affected, as signaled by the appearance of additional peaks. Since the position of these peaks depends on the value of $\alpha$, we can fine-tune the persistent current by manipulating this parameter. However, the sharpest distinction occurs when considering the spin currents. As we can observe, Rashba renders the spin currents asymmetric around zero current and, additionally, it leads to a pulse-like pattern, similar to the one we can see in Fig. 4.

In Fig. 6a,b,e,f we show the persistent currents for the parameters of Fig. 3a,b,e,f. In this case, the Rashba spin-orbit coupling is turned off, but the two MZMs are coupled to the ring. As we can observe, the results for the charge current are not dramatically altered, except for the curves being smoother in the $N=4$ case and for a secondary peak at $N=3$ as $\xi _M$ is increased to $\xi _M=0.1$. These peaks feature in a clearer fashion in the spin currents, where a small plateau for $\xi _M=0.1$ is observed at certain values of flux. This can be understood from Fig. 3. Take for instance the spectrum around $\Phi =0$ in panel (f) of that figure. Although it is very subtle, there is a switching of the Majorana states at zero energy and it is in the region between the two crossings where this plateau takes place. Additionally, we can observe that there is no suppression of the spin current upon increasing $\xi _M$, as there was when $\lambda _2=0$. Therefore, we can see that, by coupling the ring to the two MZMs we are able to suppress spin current for a range of fluxes. This could be a smoking gun in the detection of MZMs as this situation is completely different from the result in the absence of MZMs (cf. Fig. 4).

Finally, Fig. 6c,d,g,h corresponds to the case shown in Fig. 3c,d,g,h. In this scenario, the currents are similar to those of panels (a), (b), (e) and (f), but with the additional peak that was observed for $N=4$ in Fig. 5c, although clearly smoothed out. In fact, the additional peak for the $N=3$ case is completely suppressed. This has an effect on the spin current, where the $N=4$ case still shows a pulse-like behaviour, but the $N=3$ case shows plateaus for a larger range of fluxes than in panels (a), (b), (e) and (f).

## Conclusions

MZMs stand out for their remarkable properties in the realm of topological quantum matter, such as their non-Abelian statistics. However, although significant progress has been made in the direction of their experimental detection in recent years, they cannot be confirmed as yet. More importantly, recent discoveries show that it is necessary to carefully examine different effects to determine their existence ^31^ unambiguously. In this regard, we propose to consider a topological superconducting nanowire in proximity to a quantum ring threaded by a magnetic flux. It is the natural extension of the system presented in Ref. ^17^ where a nanowire is coupled to a single quantum dot. In addition, we propose the ring to display Rashba spin-orbit coupling. As we have shown in this paper, the BdG spectra are entirely altered by coupling the quantum ring to one or two MZMs and having a nonzero Rashba spin-orbit coupling. The consequences are clearly observed in the persistent currents. Interestingly, spin-orbit coupling leads to an asymmetric spin current with a square pulse-like behavior. Moreover, the coupling to the two MZMs leads to plateaus for odd-numbered rings due to the switching of MZMs at zero energy. We believe that our work could serve as an additional signature towards probing the existence of Majorana zero modes.

## Supplementary Information

Supplementary Information.
