# Supplementary material for: Manipulation of Majorana bound states in proximity to a quantum ring with Rashba coupling
Source: Sci Rep. 2022 Jan 20;12:1071. doi: 10.1038/s41598-022-05043-y (PMC8776818; doi:10.1038/s41598-022-05043-y)
Supplement: Supplementary file 1 — Supplementary Information. [file 41598_2022_5043_MOESM1_ESM.pdf]

Supplemental Material: Manipulation of Majorana bound states in proximity to a quantum ring with Rashba coupling

Fabián Gonzalo Medina[1,+,*], Dunkan Martínez[2,+], Álvaro Díaz-Fernández[3], Francisco Domínguez-Adame[2], Luis Rosales[1], and Pedro A. Orellana[1]

[1]Departamento de Física, Universidad Técnica Federico Santa María, Casilla 110 V, Valparaíso, Chile
[2]GISC, Departamento de Física de Materiales, Universidad Complutense, E–28040 Madrid, Spain
[3]GISC, GSC, Departamento de Estructuras y Física de Edificación, Universidad Politécnica de Madrid, E–28031 Madrid, Spain
[*]fmmedinacfabian@gmail.com
[+]These authors contributed equally to this work

Quasiparticle spectra, Charge and Spin Currents For $N = 5, 6$.

The spectra and currents for $N = 5, 6$ are presented.

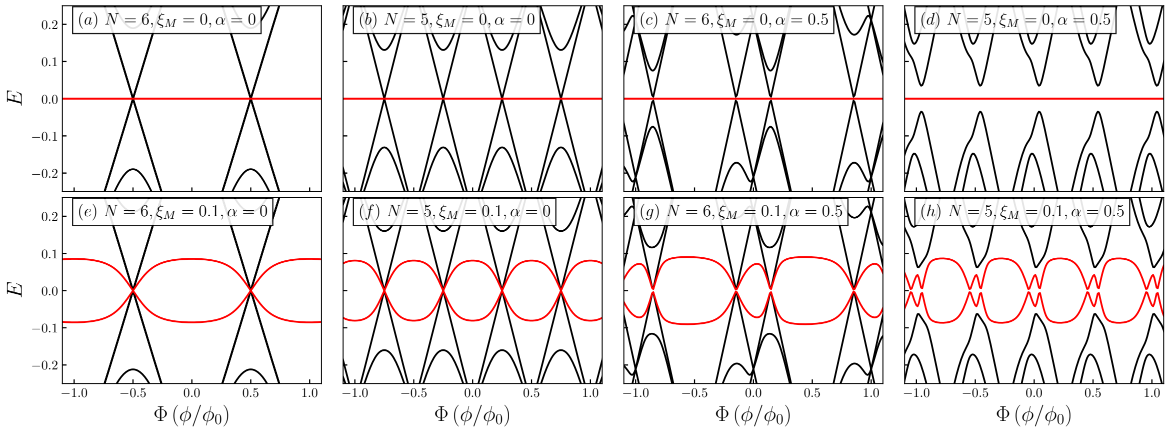

Figure 1: Quasiparticle spectra for $\lambda_1 = 0.1$ and $\lambda_2 = 0$. The number of sites, N, the coupling between MZMs, ξ_M, and the Rashba spin-orbit coupling, α, are indicated in the figure. Black curves represent the states in the quantum ring and the red curves correspond to the Majorana modes.

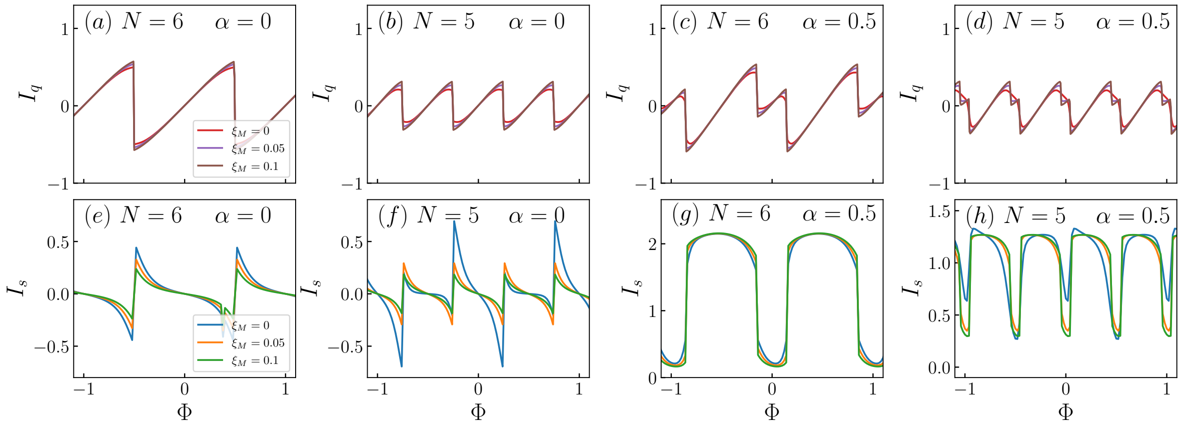

Figure 2: Charge and spin persistent currents, I_q and I_s respectively, for $\lambda_1 = 0.1, \lambda_2 = 0$. The number of sites, N, and the Rashba spin-orbit coupling, α, are indicated in the figures. The coupling between MZMs, ξ_M, is indicated by coloured lines, shown in the leftmost panels.

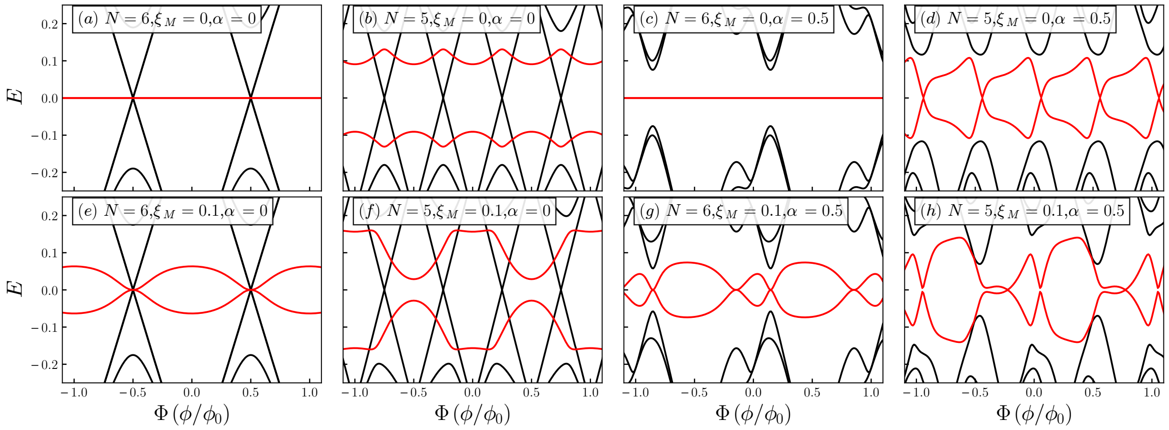

Figure 3: Quasiparticle spectra for $\lambda_1 = 0.1$ and $\lambda_2 = 0.15$. The number of sites, N, the coupling between MZMs, ξ_M, and the Rashba spin-orbit coupling, α, are indicated in the figure. Black curves represent the states in the quantum ring and the red curves correspond to the Majorana modes.

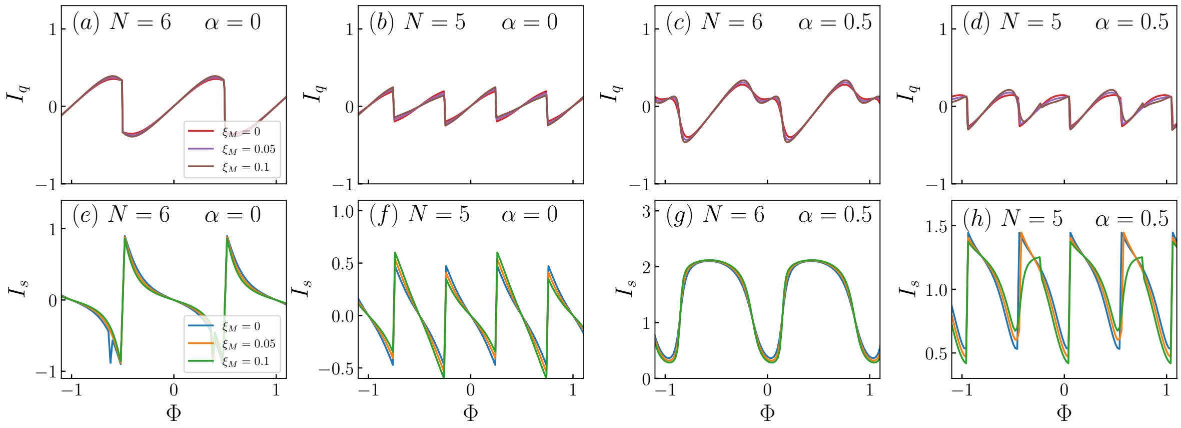

Figure 4: Charge and spin persistent currents, I_q and I_s respectively, for $\lambda_1 = 0.1, \lambda_2 = 0.15$. The number of sites, N, and the Rashba spin-orbit coupling, α, are indicated in the figures. The coupling between MZMs, ξ_M, is indicated by coloured lines, shown in the leftmost panels.

The spectra obtained for $N = 5$ show avoiding crossings between Majorana modes in panel (b) and (f) from Figure 3. In the spectra for $N = 3$ Majorana modes display zero energy crossing around 0.5 and -0.5, as we mention before, this does not happen for $N > 5$ as can be observed in panel (f) from Figure 6.

Quasiparticle Spectra for $N = 11, 12$.

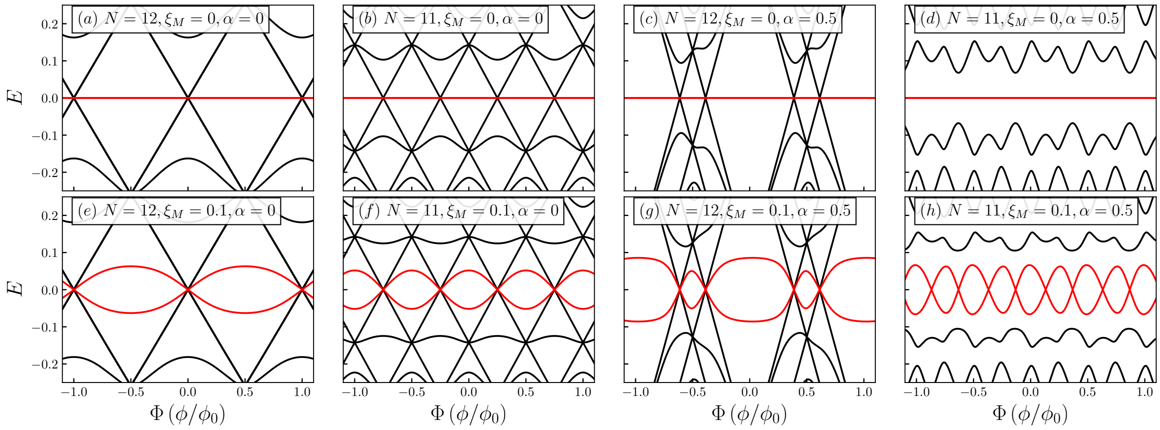

Figure 5: Quasiparticle spectra for $\lambda_1 = 0.1$ and $\lambda_2 = 0$. The number of sites, N, the coupling between MZMs, ξ_M, and the Rashba spin-orbit coupling, α, are indicated in the figure. Black curves represent the states in the quantum ring and the red curves correspond to the Majorana modes.

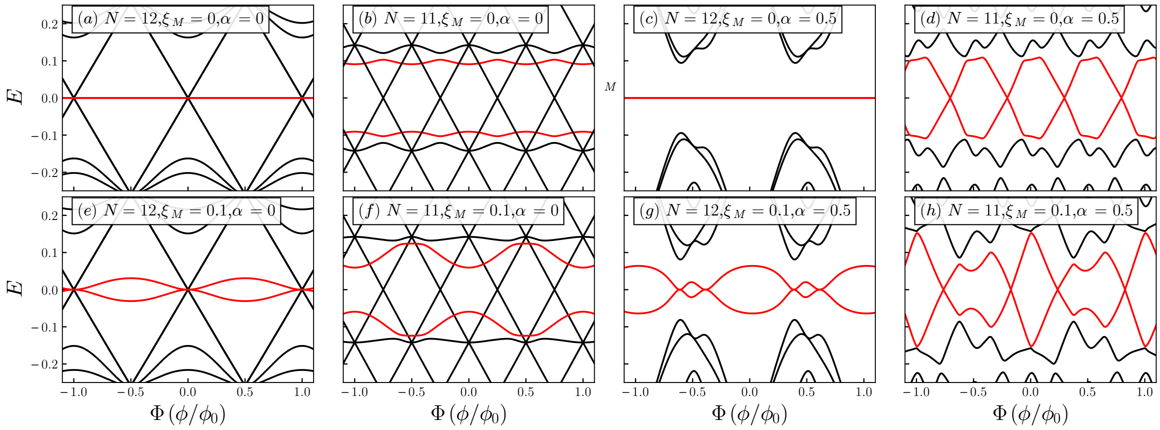

Figure 6: Quasiparticle spectra for $\lambda_1 = 0.1$ and $\lambda_2 = 0.15$. The number of sites, N, the coupling between MZMs, ξ_M, and the Rashba spin-orbit coupling, α, are indicated in the figure. Black curves represent the states in the quantum ring and the red curves correspond to the Majorana modes.
